# Congenital syphilis, syphilis in pregnancy and prenatal care in Brazil: An ecological study

**DOI:** 10.1371/journal.pone.0306120

**Published:** 2024-06-25

**Authors:** Izabelle Bezerra Costa, Isac Davidson Santiago Fernandes Pimenta, Kezauyn Miranda Aiquoc, Ângelo Giuseppe Roncalli da Costa Oliveira

**Affiliations:** 1 Post-Graduation Program in Public Health, Federal University of Rio Grande do Norte, Natal, Brazil; 2 Department of Odontology, Federal University of Rio Grande do Norte, Natal, Brazil; Universidade Federal do Rio Grande do Norte, BRAZIL

## Abstract

The aim of this research was to evaluate the incidence of congenital syphilis and the ratio between congenital syphilis and syphilis in pregnant women in Brazil according to socioeconomic indicators (inadequate water supply and sanitation; illiteracy at 15 years of age or older; household income per capita; proportion of poor people; Gini index; human development index; and average health expenditure per inhabitant by the health system) and prenatal quality-of-care indicators. We conducted an ecological study using a sample composed of 257 municipalities, each with ≥ 100,000 inhabitants. Data was collected from four public databases: the Brazilian Institute of Geography and Statistics, comprising socioeconomical data from the 2010 census; and the data of 2019 available in the databases of the Department of Informatics of the Brazilian Health System, Information and Management of Primary Care, and the Electronic Citizen Information System. Descriptive analysis of dependent and independent variables and bivariate analysis by Negative Binomial regression were carried out. The mean incidence of congenital syphilis was 38% higher in municipalities with a Human Development Index up to 0.785 (ratio of means [RM] = 1.38; *p* = 0.049) and 57% higher among populations where less than 50% of primary healthcare services provided a rapid test for syphilis (RM = 1.57; *p* < 0.001). The ratio between congenital syphilis and syphilis in pregnant women was 29% higher in municipalities with a low household income per capita (RM = 1.29; *p* < 0.001) and 28% higher in locations where less than 50% of the primary healthcare services provided a rapid test for syphilis (RM = 1.28; *p* < 0.001). There was no statistical significance of the quality of prenatal care compared to the outcomes. This result underscores the challenges in detecting syphilis infections among pregnant women during prenatal care, consequently increasing the risk of vertical transmission of the disease to the fetus. Traits of inequality in the occurrence of congenital syphilis also draw attention to strategies to reduce health inequities and improve prenatal care.

## Introduction

Syphilis is characterized as a serious sexually transmitted systemic infection caused by the spirochete *Treponema pallidum*. During pregnancy, when affected by the pathology, the pregnant woman can transmit the infection to the fetus, resulting in serious maternal and child outcomes in most of the untreated or inadequately treated cases [1,2].

Despite congenital syphilis being preventable and with low-cost treatment, the disease still represents a major public health challenge [3–5]. According to the Pan American Health Organization (PAHO), in 2017 about 29,000 cases of congenital syphilis were reported in the American countries, with an incidence rate of 2.1/1000 live births. Among the reported cases, 85% corresponded to Brazil, which had a national rate of 8.5/1000 live births [6].

This may be explained by the fact that unlike other American countries, in Brazil, it has been mandatory since 2010 to report syphilis cases of any type [6]. Additionally, the expansion in Primary Health Care (PHC) coverage and the diagnostic capacity for syphilis have facilitated the early detection of the disease and improved the quality of epidemiological information and investigation [6,7]. Nevertheless, despite these conditions, it is noticeable that syphilis rates have grown significantly in the country [8].

The prenatal period is the prime time for prevention and control of the disease in pregnant women, ultimately preventing transplacental spread of the etiological agent [2–6,9,10], and is an essential moment to provide qualified care, especially to vulnerable population groups whose social, economic and racial implications act as barriers for adherence to treatment [10]. However, the literature highlights important inequality in access to prenatal care, related to conditions such as race, years of study and living outside of urban centers [8,11–14].

In Brazil, failures related to prenatal care have shown their effects on the health of newborns. An ecological study indicated that congenital syphilis and other congenital infections are responsible for the highest proportion (29.5%) of newborn hospitalizations caused by primary healthcare-sensitive conditions [14].

In 2011 the Brazilian Ministry of Health established the Stork Network (SN; Rede Cegonha in Portuguese). In integration with preceding health policies, SN emerges as an important national strategy for improving maternal and childcare. Its proposal is based on enabling access to family planning, offering care throughout the pregnancy-postpartum cycle for women, ensuring safe childbirth, monitoring the development of children and reduce infant mortality with a focus on newborns (children up to 28 days old) [15,16].

Adherence to the program by the Brazilian municipalities could be for all levels of care or for just one level–prenatal care for example. Adherence to the program could also be made by a group of municipalities in the same region (regional adherence). To evaluate its implementation, a series of strategic indicators were established [16].

In contrast to implementation of the SN by the Ministry of Health, elevated rates of gestational syphilis and congenital syphilis persist even after a decade of implementing the network. Given this high number of cases, important questions arise about their relationship with the reporting of cases and the quality of prenatal care [6,8].

It is well known that obstetric and neonatal care improved after the implementation of SN. Studies have shown a reduction in maternal and neonatal mortality, and an increase in the number of prenatal care consultations [17–19]. However, according to a study carried out in the state of Piauí, of the four components that compose the SN (I—Pre-Natal; II—Childbirth; III—Postpartum and Comprehensive Child Health Care; and IV—Logistics, Health Transport and Service Regulation), the prenatal care component continues to face challenges, recording low proportions of women being tested for sexually transmitted infections and continuity of care [20]. This is reflected in the high rates of infection of syphilis infection among pregnant women that are not diagnosed and/or treated adequately, leading to a high number of congenital syphilis cases.

It is evident that the fragility in the SN is also attributed to insufficient monitoring and inadequacies in the evaluation process of planned actions, low adherence to the utilization of information systems established by the Health Ministry, lack of coordination among network services within local health systems and across municipalities, insufficient publicity of services and actions provided, and limited awareness of the internal flow of health regions among healthcare professionals [20].

To understand the conditions that lead to such a high incidence of this disease, it is necessary to correlate the incidence of congenital syphilis and syphilis in pregnant women to the adherence to quality care practices established by the SN prenatal component, while also considering the influence of socioeconomic conditions.

However, the few studies conducted in this sense focused only on one Brazilian state or in a few municipalities. No national research was found that quantitatively evaluated the effects of adherence to the prenatal component of the SN and its relationship with the incidence of congenital syphilis [21,22].

In this sense, the aim of this study was to evaluate the relation between the incidence of congenital syphilis and syphilis in pregnant women in Brazil and a series of socioeconomic indicators and the quality indicator of prenatal care proposed by the SN.

## Methods and analysis

This is an ecological study conducted with secondary data available in the information systems of the Brazilian Ministry of Health.

### Sampling plan

For development of the study, we consider cities with a population ≥ 100,000 inhabitants in a universe of 5570 Brazilian municipalities [23]. We opted to analyzed larger municipalities considering their homogeneity in terms of financing, management and structure of healthcare system and demographic characteristics [24].

In total, 286 cities that joined the SN were selected, according to data made available by the Ministry of Health via the Electronic Citizen Information System [25]. We do not make a distinction between the regionalized adherence modality and adherence only to the prenatal component, as we understood that the type of adherence would not influence the results of this research, considering that all Brazilian municipalities had adhered to the prenatal component, and regional adherence would provide additional services and logistical support for childbirth care.

### Data collection

Secondary data collection was carried out between May and July 2021 from the following databases: (a) Brazilian Institute of Geography and Statistics (IBGE)–a public database comprising socioeconomical data about the municipalities; (b) Department of Informatics of the Brazilian Health System (DATASUS)–a public database of the Brazilian National health system that comprise data about a variety of health information at the populational level, encompassing the incidence of diseases for which notification is mandatory, such as congenital syphilis; (c) Information and Management of Primary Care (e-Gestor)–a public database used for monitoring indicators related to primary healthcare, including those related to the SN; and (d) Electronic Citizen Information System (e-SIC)–a web portal for requesting information about any public policy or government activity. We utilized this portal to request information for verifying municipalities’ adherence to the SN.

For this analysis, we exclusively employed data up to the year 2019. This time frame predates the COVID-19 pandemic, when the data from the Brazilian Ministry of Health were updated. The sociodemographic data used as reference were from 2010, year of the last census fully available in the public databases at the time.

### Variables

Considering the prenatal period as a crucial time for the prevention of vertical transmission of syphilis and other diseases, we selected only variables related to this period. These were subdivided into five major groups: morbidity indicators; socioeconomic indicators; care quality indicators of the SN; process indicators of the Prevent Brazil Program; and additional indicators. We present descriptions, formulas and data origin and year of correspondence for all these indicators in Table 1.

**Table 1:** Descriptions and formulas for the socioeconomic and prenatal care indicators.

Indicator	Description	Formula	Font and year of reference of the data
***Dependent variables***			
Incidence of congenital syphilis	The number of cases of congenital syphilis detected in children < 1 year of age for every 1000 live births in the geographic space in a given period	Number of new cases of congenital syphilis in children <1 year of age in a given year of diagnosis and place of residence × 1000 / Total number of live births to mothers residing in the same place in the year considered	DATASUS database -2019
Ratio of congenital syphilis/syphilis in pregnant women	Ratio between the number of cases of congenital syphilis detected in children < 1 year of age in the geographic space in a given period and the number of cases of syphilis detected in pregnant women in the geographic space in a given period	Number of cases of congenital syphilis detected in children < 1 year of age in the geographic space in a given period / Number of cases of syphilis detected in pregnant women in the geographic space in a given period	DATASUS database -2019
***Independent variables***			
Proportion of people living with inadequate water supply and sanitation	Percentage of the resident population inadequately served by water supply and sanitary sewage in the general supply network, with or without domestic plumbing, in a given geographic space in the year considered	(Population residing in permanent private households inadequately served by a general water supply and sewage system, with or without internal plumbing) / (Total population of residents in permanent private households, adjusted for mid-year) × 100	IBGE database -2010
Illiteracy of people aged ≥ 15 years	Percentage of people aged ≥ 15 years who cannot read and write at least one simple note, in the language they know, in the total resident population of the same age group in a given geographic space in the year considered	(Number of residents aged ≥ 15 years who cannot read and write a simple note in the language they know) / (Total resident population in this age group) × 100	IBGE database -2010
Household income per capita	Ratio of all monthly income of a family to the number of family members in BRL (R$)	Sum of all income of a family / Number of family members	IBGE database -2010
Proportion of poor people	Percentage of the resident population with monthly per capita family income of up to half the minimum wage* in a given geographical space in the year considered	Resident population with monthly per capita family income of up to half the minimum wage) / (Total resident population) × 100	IBGE database -2010
Gini index	Index that measures the degree of concentration of the per capita household income distribution of a given population in a given geographic space	It is calculated as a ratio of the areas in the Lorenz curve diagram: Gini = Area of concentration / Area of perfect inequality	IBGE database -2010
Human Development Index (HDI)	Index of summary measure of long-term progress in three basic dimensions of human development (income, education and health) of a given population in a given geographic space	Average weighting between income, education and health of a given population in a given geographic space	IBGE database -2010
Health expenditure per inhabitant	Public spending on health per inhabitant, according to the sphere of government, in a given geographic space in the year considered, in BRL (R$)	Value of public expenditure on health / Total resident population, adjusted for mid-year	IBGE database -2016
Family Health Strategy coverage	Proportion of the population of the federated unit served by Family Health Strategy teams	Number of Family Health Strategy teams implemented × 3000 / Resident population × 100	E-Gestor database—2019
Proportion of primary healthcare services with rapid test for syphilis available	Percentage distribution of availability of rapid tests for syphilis in primary healthcare services	(Primary healthcare services that have rapid tests for syphilis) / (Total primary Healthcare services) × 100	E-Gestor database—2019
Pregnant women with 1st prenatal consultation per number of live births	Ratio between the number of pregnant women with the first prenatal care performed in a given geographic space in the considered year and the number of live births in a given geographic space in the considered year	Ratio between the number of pregnant women with the first prenatal care performed in a given geographic space in the considered year / Number of births in a given geographic space in the considered year	E-Gestor database—2019
Proportion of pregnant women with 1st consultation in the first 12 weeks	Percentage distribution of women who started prenatal care early in the first trimester of pregnancy (up to the 12th week of pregnancy)	(Number of pregnant women starting prenatal care up to the 12th week of pregnancy in a given period and location) / (Total pregnant women registered in the period and location) × 100	E-Gestor database—2019
Proportion of pregnant women submitted to routine tests up to the 20th week	Percentage distribution of pregnant women monitored during prenatal care who received routine hemoglobin, hematocrit, glycemia, urine culture, VDRL and HIV tests and received the results by the 20th week of pregnancy	(Number of pregnant women, followed up during prenatal care who underwent the routine tests and received results by the 20th week of pregnancy in a given period and place) / (Total pregnant women followed up in the same period and place) ×100	E-Gestor database—2019
Proportion of pregnant women with at least six consultations	Percentage distribution of pregnant women who had six or more prenatal consultations	(Number of pregnant women with six or more prenatal consultations in a given place and period) / (Total number of pregnant women followed up in the same place and period) × 100	E-Gestor database—2019
Proportion of pregnant women with six consultations, 1st–20th week	Proportion of pregnant women who had the number of prenatal consultations recommended by the Ministry of Health (six consultations), with the first carried out by the 20th gestational week, in relation to the total number of pregnant women in the municipality	Number of women with pregnancies completed in the period, registered, identified and linked correctly to a primary healthcare team with at least six consultations of prenatal care, where the first consultation carried out has a difference of at most 20 weeks from the first day of the last menstrual period registered in the service / Number of pregnant women registered, identified and correctly linked to the primary healthcare team with completed pregnancies (considering the probable date of delivery + 14 days) in the period	E-Gestor database—2019
Proportion of pregnant women tested for HIV and syphilis	Proportion of pregnant women who underwent syphilis and HIV tests during prenatal care performed in primary healthcare (serology evaluation and rapid test performed) in relation to the total estimated pregnant women in the municipality	Women with pregnancies completed in the period, registered, identified and linked to a primary healthcare team who were tested with serology for syphilis and HIV (VDRL) or who underwent the rapid test for syphilis and HIV / Number of pregnant women registered, identified and linked correctly to the team with completed pregnancies (considering the probable date of delivery + 14 days) in the period	E-Gestor database—2019
Prenatal Care Quality Score	Index to assess the quality of prenatal care using a combination of indicators available in the Brazilian Ministry of Health Databases	Factorial analysis of the following variables: pregnant women with 1st prenatal consultation per number of live births; proportion of pregnant women submitted to routine tests up to the 20th week of pregnancy; proportion of pregnant women with at least six consultations; proportion of pregnant women with six consultations, 1st–20th week; proportion of pregnant women tested for HIV and syphilis	E-Gestor database—2019

* Minimum wage in Brazil in 2010 were 510 BRL.

Regarding the dependent variables, the incidence of congenital syphilis is the main epidemiological indicator of the occurrence of syphilis in newborns. The ratio of congenital syphilis to syphilis in pregnant women provides valuable information as it assesses by evaluating the effectiveness of preventive measures specifically targeted at pregnant women. A value above 1 for this indicator reveals that the strategies for identifying and treating pregnant women with syphilis are not being effective enough to prevent the disease in the newborn.

The independent variables were chosen considering that socioeconomic and care factors, especially those related to prenatal care, are important explanatory elements for the occurrence of outcomes. In the first case, variables related to sanitary conditions (water supply and sanitation), education (illiteracy), income (per capita income and poverty) and inequality (Gini index) were included, in addition to the HDI, a composite indicator that aggregates indicators for longevity, income and education. In the second case, health expenditure per inhabitant and Family Health Strategy coverage were included as general indicators of assistance. The other care variables are all related to prenatal care and have been used by the Brazilian government as indicators to evaluate the quality of care.

For the cross-sectional analysis, a “Prenatal Care Quality Score” was created from five independent variables: “Pregnant women with 1st prenatal consultation per number of live births”; “Proportion of pregnant women submitted to routine tests up to the 20th week of pregnancy”; “Proportion of pregnant women with at least six consultations”; “Proportion of pregnant women with six consultations 1st–20th week”; and “Proportion of pregnant women tested for HIV and syphilis”.

For the Prenatal Care Quality Score, we conduct a factor analysis. The aim of the factor analysis is to make association patterns resulting in linear combinations of the initial variables with different weight assignments. To conduct this analysis, we used a standardization procedure to obtain standard deviation values (*z*) of each variable included, followed by the extraction and construction of factors with attribution of weights for each of the components. In the extraction stage, 57.4% of the variance was explained by the components selected.

Subsequently, the sample adequacy measure of Kaiser-Meyer-Olkin (KMO) was calculated. The KMO index was 0.743, a value considered acceptable for the proposed objectives. Finally, the Bartlett χ^2^ sphericity test (529.9; p < 0.001) was applied to test the correlation between variables in the population. In this sense, the null hypothesis of this test is that there is no correlation between the variables and the null hypothesis is rejected when the observed chi-square is greater than that contained in the tables and has a statistical significance of 5% [26]. We did not include the variable “Proportion of pregnant women with 1st consultation in the first 12 weeks” as it presented strong collinearity in the factor analysis.

### Data analysis

Considering that the outcome variables (incidence of congenital syphilis and ratio of congenital syphilis/syphilis in pregnant women) as well as the independent variables are all quantitative, the descriptive analysis of the data was carried out by calculating the measures of central tendency and variability (mean, median and standard deviation) in addition to the minimum maximum values. Since we were working with aggregated data, in an ecological study design, all measures were weighted by the municipality’s population.

The independent variables were then categorized in order to compare the means of the outcomes for each exposure category and, thus, obtain a ratio of means as a summary measure of the effect. To obtain an adjusted analysis, Poisson modeling is the most suitable for situations like this, in which the outcomes can be considered as counting data. However, a high variability was observed, which caused an overdispersion effect. In this sense, we opted for the Negative Binomial regression, which minimizes the effects of the overdispersion, although using the same estimates as Poisson regression. The final equation of the model is as follows:

$$\ln(\mu )=\beta_{0}+\beta_{1}x_{1}+\beta_{2}x_{2}+\ldots \beta_{k}x_{k}$$

Where ln(μ) is the logarithm of the expected number of occurrences, β0 is the constant, βj (j = 1,2,… k) are the parameters for each exposure variable and Xj are the explanatory variables.

### Ethical considerations

As the data are in the public domain it was not necessary to submit a request to the Ethics and Research Committee, as provided for in Resolutions 510/2016 and 466/2012 of the National Health Council of Brazil [27,28].

## Results

Considering that the data were obtained from official databases, very few missing values were identified, not interfering in analysis. Regarding outliers, the database was initially evaluated based on normalized data, where univariate atypical data were identified. At least six variables (“proportion of pregnant women with 1st consultation in the first 12 weeks”; “proportion of pregnant women submitted to routine tests up to the 20th week”; “proportion of pregnant women with at least six consultations”; “proportion of pregnant women with six consultations 1st-20th week”; “incidence of congenital syphilis” and “ratio of congenital syphilis/syphilis in pregnant women”) presented outliers, with more than 3 standard deviations above the mean. For this reason, it was decided to use outliers multivariate analysis, based on the calculation of the Mahalanobis distance [29]. 29 multivariate outliers were then identified (10.1%), which were excluded from the sample, leaving 257 municipalities.

### Descriptive analysis

Table 2 summarizes the descriptive analysis of the dependent and independent variables, highlighting their measures of central tendency and variability weighted by the population.

**Table 2:** Dependent and independent variables and their measures of central tendency and population-weighted variability.

Variable	Mean	SD	Median	Min.	Max.
***Dependent***					
Incidence of congenital syphilis	10.7	7.9	8.6	0.0	42.3
Ratio of congenital syphilis/syphilis in pregnant women	0.4	0.2	0.4	0.0	1.1
***Independent***					
Proportion of people living with inadequate water supply and sanitation	2.4	4.6	0.7	0.0	42.2
Illiteracy of people aged ≥15 ***years***	5.2	3.4	3.9	1.6	30.5
Household income per capita (in BRL)	1030.60	388.83	950.34	226.99	2043.74
Proportion of poor people	8.2	6.4	5.3	0.5	50.7
Gini index	0.6	0.1	0.6	0.4	0.7
Human Development Index (HDI)	0.765	0.043	0.768	0.577	0.862
Health expenditure per inhabitant, 2016 (in BRL)	743.46	321.74	765.70	199.68	4.368.47
Family Health Strategy coverage	48.9	21.6	46.0	0.0	100.0
Proportion of primary healthcare services with rapid test for syphilis available	82.8	28.1	97.1	0.0	100.0
Pregnant women with 1st prenatal consultation per number of live births	44.1	21.5	45.6	0.0	93.8
Proportion of pregnant women with the 1st consultation in the first 12th weeks	7.7	18.2	1.5	0.0	100.0
Proportion of pregnant women submitted to routine tests up to the 20th week of pregnancy	5.6	5.0	3.7	0.0	25.5
Proportion of pregnant women with at least six consultations	17.8	14.7	13.3	0.0	54.0
Proportion of pregnant women with six consultations, 1st–20th week	20.9	15.6	18.7	0.0	55.3
Proportion of pregnant women tested for HIV and syphilis	19.8	16.6	15.3	0.0	73.7

SD = standard deviation.

### Cross-sectional analysis

Table 3 shows the incidence of congenital syphilis in 2019 according to the proposed variables. Through multivariate analysis with adjusted values, it was observed that only “HDI” and “Proportion of primary healthcare services with rapid test for syphilis available” showed a statistically significant relation (*p* < 0.05) with the incidence of congenital syphilis. It was found that in municipalities with an HDI up to 0.785 there was a 38% increase (ratio of means [RM] = 1.38; *p* = 0.049) in the incidence of infection. In addition, in locations with low availability of a rapid test for syphilis (up to 49.9% of the primary healthcare services), an increase of 57% (RM = 1.57; *p* < 0.001) in new cases of congenital syphilis can be seen. There was no statistical significance for the quality of prenatal care.

**Table 3:** Incidence of congenital syphilis (per 1000 live births) in 2019 in relation to independent variables.

		Incidence of congenital syphilis (x 1000 LV) 2019			Not adjusted			Adjusted		
	*n*	Mean	SD	Median	RM	95% CI	*p*	RM	95% CI	*p*
**Proportion of people living with inadequate water supply and sanitation**										
High (> 0.92%)	129	8.86	6.08	7.00	1.37	1.36–1.38	<0.001			
Low (≤ 0.92%)	128	12.17	9.71	10.00	1					
**Illiteracy of people aged ≥ 15 years**										
High (> 4.93%)	130	9.45	7.72	7.00	1.24	1.23–1.25	<0.001			
Low (≤ 4.93%)	127	11.73	8.09	10.00	1					
**Household income per capita (in BRL)**										
Low (≤ R$ 787)	128	9.33	6.37	8.00	1.31	1.30–1.32	<0.001			
High (> R$ 787)	129	12.29	10.51	10.00	1					
**Proportion of poor people**										
High (> 6.9%)	129	9.14	7.25	7.00	1.28	1.27–1.29	<0.001			
Low (≤ 6.9%)	128	11.75	8.58	10.00	1					
**Gini index**										
High (> 0.52)	143	11.40	10.64	8.00	0.84	0.83–0.85	<0.001			
Low (≤ 0.52)	114	9.59	6.03	8.00	1					
**Human Development Index**										
Low (≤ 0.785)	128	8.98	6.30	7.00	1.42	1.41–1.43	<0.001	1.38	1.02–1.90	0.049
High (> 0.785)	129	12.80	10.11	10.00	1			1		
**Health expenditure per inhabitant. 2016 (in BRL)**										
Low (≤ R$ 646)	127	9.29	6.45	7.00	1.27	1.26–1.28	<0.001			
High (> R$ 646)	128	11.81	9.83	10.00	1					
**Coverage of the Family Health Strategy**										
Low (≤ 55.6%)	128	10.52	9.19	9.00	0.95	0.94–0.96	<0.001			
High (> 55.6%)	129	10.05	7.26	8.00	1					
**Proportion of primary healthcare services with rapid test for syphilis available**										
Low (≤ 49.9)	122	8.74	6.80	7.00	1.37	1.36–1.38	<0.001	1.57	1.25–1.97	<0.001
High (> 75.0)	125	12.05	8.75	10.00	1			1		
**Quality of prenatal care**										
Low (≤ 34.5)	127	9.72	6.14	8.00	1.13	1.12–1.14	<0.001			
High (> 52.5)	127	11.00	10.05	8.00	1					
**Total**	**257**	**10.19**	**7.92**	**8.00**						

Data are population-weighted measures of central tendency and variability and ratio measures calculated by Poisson regression with robust variance. The variables "Health expenditure per inhabitant", "Proportion of primary healthcare services with rapid test for syphilis available" and "Quality of prenatal care" do not add up to 257 because of missing data. RM = ratio of means; SD = standard deviation; LB = live births.

Table 4, which gives the ratio between congenital syphilis and gestational syphilis and its relation with other variables after multivariate analysis, indicates that only “Household income per capita” and “Proportion of primary healthcare services with rapid test for syphilis available” showed statistical significance. It is observed that in regions with a low income (≤ R$ 787) there is an increase of 29% (RM = 1.29; *p* < 0.001) in the ratio between congenital syphilis and gestational syphilis. Similar to the finding in the previous table, an increase of 28% (RM = 1.28; *p* < 0.001) in the dependent variable was observed in municipalities with low availability of a rapid test for syphilis (≤ 49.9% of the primary healthcare services).

**Table 4:** Ratio between congenital syphilis and syphilis in pregnant women in 2019 in relation to independent variables.

		Ratio between congenital syphilis and syphilis in pregnant women. 2019			Not adjusted			Adjusted		
	*n*	Mean	SD	Median	RM	95% CI	*p*	RM	95% CI	*p*
**Proportion of people living with inadequate water supply and sanitation**										
High (> 0.92%)	129	0.37	0.17	0.29	1.26	1.25–1.26	<0.001			
Low (≤ 0.92%)	128	0.46	0.22	0.46	1					
**Illiteracy of people aged ≥ 15 years**										
High (> 4.93%)	130	0.37	0.17	0.30	1.36	1.35–1.36	<0.001			
Low (≤ 4.93)	127	0.49	0.23	0.50	1					
**Household income per capita (in BRL)**										
Low (≤ R$ 787)	128	0.38	0.18	0.32	1.25	1.25–1.25	<0.001	1.29	1.13–1.48	<0.001
High (> R$ 787)	129	0.48	0.23	0.46	1			1		
**Proportion of poor people**										
High (> 6.9%)	129	0.34	0.17	0.28	1.51	1.51–1.51	<0.001			
Low (≤ 6.9%)	128	0.51	0.21	0.50						
**Gini index**										
High (> 0.52)	143	0.41	0.22	0.43	0.97	0.97–0.97	<0.001			
Low (≤ 0.52)	114	0.40	0.19	0.36	1					
**Human Development Index**										
Low (≤ 0.785)	128	0.38	0.18	0.29	1.25	1.25–1.25	<0.001			
High (> 0.785)	129	0.47	0.22	0.45	1					
**Health expenditure per inhabitant. 2016 (in BRL)**										
Low (≤ R$ 646)	127	0.35	0.19	0.28	1.39	1.39–1.39	<0.001			
High (> R$ 646)	128	0.49	0.19	0.48	1					
**Coverage of the Family Health Strategy**										
Low (≤ 55.6%)	128	0.45	0.22	0.43	0.85	0.85–0.85	<0.001			
High (> 55.6%)	129	0.39	0.19	0.33	1					
**Proportion of primary healthcare services with rapid test for syphilis available**										
Low (≤ 49.9)	122	0.34	0.17	0.28	1.43	1.43–1.43	<0.001	1.28	1.11–1.47	<0.001
High (> 75.0)	125	0.48	0.20	0.49	1			1		
**Quality of prenatal care**										
Low (≤ 34.5)	127	0.38	0.19	0.29	1.19	1.19–1.2	<0.001			
High (> 52.5)	127	0.45	0.20	0.45	1					
**Total**	**257**	**0.41**	**0.20**	**0.40**						

Data are population-weighted measures of central tendency and variability and ratio measures calculated by Poisson regression with robust variance. The variables "Health expenditure per inhabitant", "Proportion of primary healthcare services with rapid test for syphilis available" and "Quality of prenatal care" do not add up to 257 because of missing data. RM = ratio of means; SD = standard deviation.

## Discussion

The results show an important relationship between the high incidence of congenital syphilis in municipalities with low HDI and less availability of a rapid test for syphilis in primary healthcare services. There is also a significant association between the ratio of congenital syphilis and syphilis in pregnant women and the low household income per capita and low availability of rapid syphilis tests. In addition, aspects related to the Prenatal Care Quality Score do not seem to interfere significantly with the incidence of infection.

From this perspective, the results on the relationship between HDI and congenital syphilis rates can be explained by the exacerbation of health inequities in poorer and, consequently, more vulnerable populations. A Brazilian study showed that low income and the vulnerability associated with poverty are also associated with obstacles such as inadequate prenatal care [30]. Other studies also point out that unemployed pregnant women or those with lower incomes and lower levels of schooling or academic training are more likely to have a child with congenital syphilis due to disparities in access to healthcare [4,13].

In one aspect, it is understood that families with low HDI and poor families have limitations in terms of access to care, being exposed to health inequities [31]. However, unlike the groups of people with greater purchasing power, the poorest strata tend to seek health services more because of illness or disability and not for reasons of prevention or routine examinations [32].

The socioeconomic indicators reflect an iniquitous scenario of deprivation of rights for a large part of the population and, as the literature points out, this impacts on the health conditions of individuals [31–33]. From this perspective, studies carried out in the Brazilian states of Pernambuco and Minas Gerais identified that having low education, having a per capita income below one minimum wage, among other factors, were directly related to both gestational syphilis and inadequate prenatal care [30,34,35]. Sociodemographic conditions also play a significant role in influencing the adherence to treatment for the disease in pregnant women. Incomplete treatment is associated with factors such as age, lower income, and fewer years of education [36]. On the other hand, factors such as better HDI, being over 21 years of age, living in large cities or close to large centers, in addition to residing in the southwest of Brazil, seem to be related to better prenatal care [37].

Another point to be noted is that, in general, there was a high availability of rapid tests for syphilis in the context of primary healthcare, demonstrating the potential of municipalities for testing pregnant women. Nevertheless, a low proportion of pregnant women were tested for HIV and syphilis.

A study with national data from 2012 to 2018 observed an increase in the performance of rapid tests for syphilis in pregnant women in primary healthcare, which culminated in an increase in the number of reports of gestational syphilis cases [7]. Data from the Brazilian Ministry of Health indicate that in 2019 alone, 12,165,070 rapid tests for syphilis were distributed, which is much higher than in previous years: for example, in 2011 the distribution was only 31,500 rapid tests. However, even in the face of this advance, the authors draw attention to the insufficient number of tests performed on pregnant women during the studied period [7].

The results in this study did not find a correlation between a high Prenatal Care Quality Score and a lower incidence of congenital syphilis. This can be explained by the fact that the indicators that constitute the index, such as first prenatal consultation, early recruitment of pregnant women and minimum number of consultations mainly refer to accessibility to the services and do not consider another quality dimensions such as effectiveness, equity, integration of services and timeliness [10].

Nevertheless, we can infer that a low proportion of consultations during prenatal care and a low rate of tests performed in pregnant women may have an important influence on the higher incidence of congenital syphilis and syphilis in pregnant women, especially when the test is not available in primary healthcare services.

A global survey showed that factors such as coverage of the first prenatal consultation, availability of rapid tests and provision of treatment for pregnant women with syphilis had better scores over the years–coverage of the first prenatal consultation, for example, increasing from 85% in 2012 to 88% in 2016. This same study showed that negative outcomes during pregnancy (including vertical transmission of syphilis) were associated with not performing prenatal care (21%), access to prenatal care but not offering tests (57%), application of tests but no treatment (16%) and application of tests but without adequate treatment or with the occurrence of reinfection (6%) [38].

Considering these factors, it is highly recommended that investigation of syphilis in pregnant women at the beginning of pregnancy, and from the 28th week of pregnancy in more vulnerable groups [2,39,40], is preferably through the rapid test, which, in the face of an infection, is more sensitivy [40].

The literature also points out the importance of access to prenatal care in the prevention and control of sexually transmitted diseases in pregnant women in order to prevent the transplacental transmission of diseases and the occurrence of negative outcomes in newborns [2,41]. Factors such as adequate testing of pregnant women and administration of adequate pharmacological treatment for infected women and partners during prenatal consultations are strategies that are recognized as effective and consensual among researchers in the reduction of congenital syphilis [2,11,30,38,39,42].

Such activities are under the responsibility of healthcare professionals at the primary care level. A study conducted in the city of Teresina, capital of Piaui, a Brazilian Northeast State, investigated the knowledge and practices of primary care health professionals regarding the management of syphilis during pregnancy. The findings revealed that approximately 30% of the participants had never received training in syphilis management at the primary care level, and only 3.8% reported the use of rapid tests for syphilis. Furthermore, 35.2% were unaware that treatment with penicillin could be administered at the primary care level, often referring pregnant women to another level of care [43]. In light of these findings, it is imperative to provide training for these professionals in the identification and therapeutic management of the infection, particularly among doctors and nurses. This training is a crucial component of the prevention and control of syphilis in its early stages [35,44,45].

Additionally, it is recognized that public campaigns aimed at raising awareness about the prevention and testing for sexually transmitted infections play a crucial role in controlling this type of disease [46]. In 2017, the Brazilian Ministry of Health released the Agenda for Strategic Actions to Reduce Syphilis in Brazil, which includes a component focused on public campaigns. These campaigns, related to the "Syphilis No" project, successfully generated public interest in syphilis prevention [47]. Furthermore, it is equally important to develop programs that raise awareness about sexually transmitted infections targeting women at early stages of pregnancy, particularly at the community level through primary care [48–50].

Considering the results of this study, despite the importance of the SN for improving the quality of prenatal care in Brazil, congenital syphilis and syphilis in pregnant women remains an important challenge to be addressed by public health stakeholders. Although one of the proposals of the program is to reduce the incidence of avoidable diseases such as syphilis, there are still problems with the accessibility of this service and the provision of effective care.

In 2022, the Brazilian government proposed a new program as a substitute for the SN–the Maternal and Child Care Network. Despite some criticism about the implementation of this program, mainly about childbirth care, the test for sexually transmitted diseases was presented as a priority in prenatal care but also at the Family Planning stage [51]. Future studies will be necessary to understand whether this program will reduce the incidence of congenital syphilis and syphilis in pregnant women.

Understandably, this study has some limitations. The quality of secondary data can interfere in the analysis. To mitigate this problem, the period for conducting the analysis was chosen so that all the revisions to the databases were already included, and we also conducted an analysis of the outliers for all the variables included.

We also did not conduct a subanalysis by Brazilian region; nevertheless, this study provides broad coverage of congenital syphilis and syphilis in pregnant women in Brazil. It is important to acknowledge that this study focuses exclusively on medium and large municipalities, characterized by enhanced healthcare infrastructure for their residents. Therefore, it is possible that the findings presented in this research may not fully capture the situation in smaller municipalities.

## Conclusion

The findings of this study portray a worrying national scenario. The low HDI showed an important correlation with the incidence of congenital syphilis, while the low household income per capita in the cities studied was related to a higher ratio between congenital syphilis and gestational syphilis. The low offer of a rapid test for syphilis was correlated with a higher incidence of congenital syphilis and a higher ratio between congenital syphilis and gestational syphilis. The quality of prenatal care offered, measured mainly by accessibility indicators, was not correlated with any of the outcomes studied.

Future studies should focus on a subanalysis of these indicators across the Brazilian regions, as there are important differences in socioeconomic development in the country. It is also important to investigate interventions that improve the quality of prenatal care in process indicators, such as testing for sexually transmitted infections across the prenatal period. The distribution of rapid tests should also consider the vulnerability of the community in each municipality.
